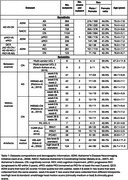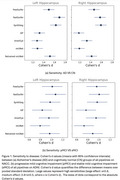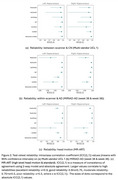# Evaluating the sensitivity and reliability of hippocampal segmentation for dementia using deep learning

**DOI:** 10.1002/alz70856_102219

**Published:** 2025-12-25

**Authors:** Jiongqi Qu, Sophie A. Martin, Anna Schroder, Matthew Grech‐Sollars, Carole H Sudre, James H. Cole

**Affiliations:** ^1^ University College London, London, United Kingdom

## Abstract

**Background:**

Deep learning is commonly used for automated hippocampal segmentation from structural MRI in dementia research. However, many studies focus primarily on segmentation accuracy. This study aims to evaluate sensitivity to disease and test‐retest reliability, which are key to clinical readiness, of automated segmentation tools.

**Method:**

We compared five pipelines (FastSurfer (Henschel et al., 2020), SynthSeg (Billot et al., 2023), Geodesic Information Flows (GIF) (Cardoso et al., 2015), InnerEye (Schroder et al., 2024), and two versions of nnUNet (Isensee et al., 2021)) based on group‐level sensitivities (Alzheimer's disease VS cognitively normal, and progressive VS stable mild cognitive impairment), and reliability using within‐scanner, between‐scanner, and intentional motion artefacts datasets, benchmarked against FreeSurfer 7.4 (Fischl, 2012). We used T1‐weighted MRI scans from the Alzheimer's Disease Neuroimaging Initiative (*n* = 2299) and the National Alzheimer's Coordinating Center (*n* = 1852) with matched age and sex for the sensitivity study, and 8 independent datasets (1264 scans) for the reliability study. Detailed information is provided in Table 1. To ensure fair comparison and align preprocessing, the nnUNet was retrained using 366 scans from the Open Access Series of Imaging Studies (LaMontagne et al., 2019).

**Result:**

For sensitivity to Alzheimer's, all pipelines showed large effect sizes (Cohen's d ≥0.8) on ADNI and NACC. FastSurfer and SynthSeg achieved the highest Cohen's d values, and nnUNet had performance comparable to FreeSurfer after retraining (Figure 1.a). Cohen's d values dropped but the ranking of pipelines remained consistent on the pMCI VS sMCI (Figure 1.b) and QC3 (sensitivity comparisons using low‐quality scans). For test‐retest reliability, while all pipelines had high mean intraclass correlation coefficient (ICC(2,1)) values on Multi‐vendor UCL 1, the 95% confidence interval ranges skewed largely to the lower bounds. The ICC(2,1) values of FastSurfer, SynthSeg, GIF, and nnUNet were substantially higher than FreeSurfer on within‐scanner datasets (Figure 2.b). GIF and InnerEye were less reliable on MR‐ART (Figure 2.c).

**Conclusion:**

This study analysed the sensitivity and reliability of 5 mainstream pipelines for automated hippocampal segmentation, benchmarked against FreeSurfer, and provided a workflow to evaluate pipelines’ clinical readiness. The strategy can also be applied to design/tailor pipelines that are robust to artefacts and scan variability.